# Transcortical photothrombotic pyramidotomy model with persistent motor deficits

**DOI:** 10.1371/journal.pone.0204842

**Published:** 2018-12-31

**Authors:** Hanlim Song, Jongwook Cho, Sunwoo Lee, Ji-Young Park, Byung-Moon Choi, Min Sun Kim, Weon Gyeong Kim, Min-Cheol Lee, Hyoung-Ihl Kim

**Affiliations:** 1 Department of Biomedical Science and Engineering, Gwangju Institute of Science and Technology, Gwangju, Republic of Korea; 2 Department of Anesthesiology and Pain Medicine, Asan Medical Center, University of Ulsan College of Medicine, Seoul, Republic of Korea; 3 Department of Physiology, Wonkwang University School of Medicine, Iksan, Republic of Korea; 4 Department of Nursing, Nambu University, Gwangju, Republic of Korea; 5 Department of Pathology, Chonnam National University Medical School, Gwangju, Republic of Korea; 6 Department of Neurosurgery, Presbyterian Medical Center, Jeonju, Republic of Korea; University of Münster, GERMANY

## Abstract

Traditional pyramidotomy models have a high mortality rate from breathing difficulties and show early recovery from the induced motor deficits. This study establishes a novel pyramidotomy technique in Sprague Dawley rats that generates persistent motor deficits and has a reduced mortality rate. We used viral neural tracing to identify the course and relative distribution of forelimb and hindlimb motor fibers (*n* = 9). On basis of the neural tracing data, the medullary pyramid was targeted dorsally from the cerebellar cortex for photothrombotic infarct lesioning (*n* = 18). The photothrombotic technique selectively destroyed the corticospinal fibers in the medullary pyramid with relative preservation of neighboring grey-matter tissue. MicroPET imaging using 2-deoxy-2-[^18^F]-fluoro-D-glucose (FDG-microPET) showed a decrease in regional cerebral glucose metabolism (rCGM) in the bilateral pyramid and ipsilateral sensory cortex (*p* < 0.001, FDR *q* < 0.05). In addition, the trapezoid bodies and superior olivary nuclei showed a decrease in rCGM, compatible with damage caused during the introduction of the optical fiber. Connected structures such as the inferior colliculi and auditory cortices also showed decreases in rCGM in both hemispheres (*p* < 0.001, FDR *q* < 0.05). There was a significant and persistent decrease in motor and sensory function in the contralateral limb following pyramidotomy, as demonstrated by performance in the single pellet reaching task and the foot-fault test. There was no operative mortality or loss of respiratory function in this study. These results indicate that photothrombotic pyramidotomy with a dorsal transcortical approach is a safe and reliable technique for generating a pyramidotomy model with persistent motor deficits.

## Introduction

The medullary pyramid is an important structure located superficially in the ventral side of the medulla oblongata. The corticospinal tract (CST) descends through the medullary pyramid to convey primary motor commands for skilled movements to the spinal cord. Occlusion of the anterior spinal artery can lead to infarctions in the medullary pyramid, the medial lemniscus, and the hypoglossal nerve, resulting in medial medullary syndrome. The medullary pyramid is also often involved in unilateral brain-stem strokes caused by large artery infarcts[1]. Medullary pyramidotomy (pyramidotomy) has been used to study the role of the CST in skilled movements[2], axonal remodeling and brain plasticity in unlesioned sections of the CST[3, 4], and recovery mechanisms from white-matter stroke[5, 6]. Considering the paucity of white matter stroke models and the different pathogenesis and recovery mechanisms involved in cortical grey-matter and subcortical white-matter stroke, a pyramidotomy model has the potential to expand stroke research beyond grey-matter stroke[7–9].

Because the brainstem contains critical structures for respiration and cardiovascular control, the medullary pyramid is typically exposed for pyramidotomy using a ventral approach; it is assumed that a dorsal approach from the cortex to the pyramid would damage these critical structures. In the ventral approach, however, it is necessary to retract the deep cervical muscles and trachea to reach the base of the skull (clivus), which can cause severe breathing difficulties and inflammation of the airway, leading to a reduced survival rate in experimental rats. After exposing the medullary pyramid, the exposed pyramid is cut under direct visual inspection. However, the extent of the cut is variable even in experienced hands because the posterior border of the pyramid is invisible and resectioning relies on individual experience. Consequently, complete and uniform lesioning of the pyramid is very challenging, even for an expert[2]. Furthermore, ventral pyramidotomy is likely to damage vessels in the vicinity, including the basilar artery and its transverse branches on the pyramid.

We have previously reported selective lesioning of the white matter in a circumscribed area of the internal capsule with preservation of the neighboring grey matter, using a technique that exploits the differential scattering of light in grey versus white matter during photothrombotic lesioning[7, 10]. It is possible to selectively destroy the former with preservation of the latter if the intensity of the light has a low enough irradiance. In this experiment, we applied this technique to create a photothrombotic infarct lesion in the medullary pyramid. To validate the technique, we assessed the size of the lesions and evaluated motor and sensory behavior. In addition, we performed serial FDG-microPET scans to assess the effect of pyramidotomy on neighboring structures and also monitored possible complications during and after pyramidotomy.

## Materials & methods

### Experimental animals

Animal experiments were performed according to GIST (Gwangju Institute of Science and Technology) institutional guidelines and all procedures for this research were approved by the Institutional Animal Care and Use Committee at GIST (GIST-2017-083). All surgery was performed under inhalation anesthesia with isoflurane gas or intramuscular injection of ketamine hydrochloride and xylazine, and all efforts were made to minimize suffering. ARRIVE guidelines were followed in the preparation of the manuscript.

In this study, we used 39 male Sprague Dawley rats (Orient Bio, Gapyung, Korea), bred purely for research purposes. The animals weighed 280g – 300g upon arrival and were approximately 8 weeks old. All the rats were housed two per cage in a controlled animal facility at 21 ± 1°C and relative humidity (45%-65%) with water ad libitum. The animal care unit was maintained on a 12-hour light-dark cycle with lights on at 6:00 am. Nine naive rats were used for neural tracing of the corticospinal tract. Experimental rats underwent photothrombotic infarct lesioning in the medullary pyramid. Eight rats were selected as the “pyramidotomy group” (n = 8) on the basis of lesion extent and behavioral performance. To be included in this group, rats needed to have unilateral destruction of the entire breadth of the medullary pyramid (with no invasion of the lesion into the contralateral side) and a 50% decrease in behavioral performance compared with their pre-lesion score. Ten rats were also selected to form a “sham-operated group” (n = 10). In addition, we performed serial FDG-microPET scans in twelve rats: six sham-operated rats and six rats in the pyramidotomy group.

### Virus injection for neural tracing of the corticospinal tract

We employed a previously described viral neural-tracing technique to map the distribution of pyramidal fibers (n = 9)[10]. Briefly, we injected adeno-associated virus serotype 5 (AAV5)-CamKIIa-EYFP and AAV5-CamKIIa-mCherry into the forelimb and hindlimb areas of the motor cortex, respectively. Rats were anesthetized with an intramuscular injection of ketamine hydrochloride (100 mg/kg) and xylazine (7 mg/kg), then head-fixed in a stereotactic frame. Two small holes were drilled through the skull above the forelimb areas of motor cortex (AP +2.0 from bregma; ML ±2.5 from the midline) and hindlimb (AP −2.0 from bregma; ML ±1.5 from the midline). One microliter of AAV virus was stereotactically injected into layer 5 of the motor cortex at a rate of 0.1 ml/min with a 30G Hamilton syringe connected to a UltraMicroPump (WPI, Sarasota, FL, USA). After injection, we waited an additional 10 minutes before slowly retracting the needle. After waiting 4 weeks for viral expression, the rats were sacrificed via cardiac perfusion with 4% paraformaldehyde solution. Brains were cut into 40 μm coronal sections. Four coronal sections were selected to observe the course and distribution of forelimb and hindlimb pyramidal fibers: at the posterior limb of internal capsule (PLIC: bregma −3.3 mm), at the caudal cerebral peduncle (bregma −6.9 mm), at the longitudinal fasciculus of the pons (LFP: bregma −8.0 mm), and at the medullary pyramid level (bregma −10.9 mm). Images were acquired with a DM3000 microscope (Leica, Germany) and iSolution DT Software (OEM-optical.com, Roseville, CA, USA). EYFP expression (forelimb motor fibers) and mCherry expression (hindlimb motor fibers) were manually drawn onto the stereotactic atlas to show the relative distribution of pyramidal fibers at each level.

### Photothrombotic lesioning in medullary pyramid and histological verification

On the basis of the neural tracing results, we performed photothrombotic infarct lesioning in the medullary pyramid (*n* = 8) with careful respiratory monitoring, according to a previously described technique[7,10]. Briefly, rats were head-fixed in a stereotactic frame under anesthesia. After drilling a small hole and removing the dura, an insulated optical fiber was gently inserted at the target (AP −9.24 from bregma, ML ±0.9 from the midline, DV −10.6 from the skull surface). This stereotactic target was chosen carefully to ensure inclusion of the medial portion of the pyramid. While body temperature was maintained at 37 ± 0.5°C, rose bengal dye (20 mg/kg) was injected through the tail vein and the target area was irradiated for 3 minutes by 532-nm green laser light with an intensity of 4.0 mW at the tip of the optical fiber. The photothrombotic procedure had a ~50% success rate in producing an infarct lesion confined to a unilateral pyramid that produced a persistent motor deficit. The sham-operated group (*n* = 10) received identical treatment except that normal saline was injected instead of rose bengal dye. After three weeks of behavioral testing, animals were anesthetized with an intramuscular injection of ketamine hydrochloride (300 mg/kg) and trasncardially perfused with 4% paraformaldehyde. Serial coronal sections were Nissl or hematoxylin–eosine (H&E) stained to measure the extent of the lesions. In addition, the sections were immunostained for glial fibrillary acidic protein (GFAP), microglia antibody (1ba1), and neurofilament protein (NF). Infarct volumes were measured in serial slides in ImageJ software (NIH, Bethesda, MD, USA).

### Anesthesia under respiratory monitoring

Two parameters of respiration, respiratory rate and end-tidal CO_2_ concentration (EtCO_2_), were used for respiratory monitoring in sham-operated (n = 10) and pyramidotomy group (n = 8). Animals were briefly anesthetized in an induction chamber filled with isoflurane gas and then head-fixed in a stereotactic frame. Respiratory rate (RR) was recorded with a force-displacement transducer (FT03, Grass Instrument Company Quincy, MA, USA). The recorded data were digitalized and monitored using data analysis software (ADinstruments, Sydney, Australia). End-tidal carbon dioxide (EtCO_2_), the partial pressure of CO_2_ at the end of an exhaled breath, was also monitored continuously with patient monitor(GE Healthcare United Kingdom, Chicago, Illinois, USA) to evaluate the effect of pyramidotomy on respiratory function. All the data were transferred to personal computers using RS232C cables. Respiratory monitoring was performed before infarct lesioning, during photothrombosis, and after lesioning (1, 5, 10, and 30 min).

### Behavioral evaluation

Two types of behavioral tests were performed in sham-operated and pyramidotomy group (n = 18) to evaluate the motor and sensory deficits due to pyramidotomy: a single-pellet reaching task (SPRT) to evaluate skilled forelimb movements, and a foot-fault task to test forelimb and hindlimb function. The SPRT was performed as previously described. A food-restricted rat was placed inside a Plexiglass box (40 cm ×45 cm × 13 cm) and trained to reach for a sucrose pellet (Bio-Serve, Frenchtown, NJ, USA) through the a 1-cm wide slit before (~1 week) and after (5 days/week for 3 weeks) pyramidotomy. Animals were given 20 pellets per session. A successful reach was defined as a reach in which an animal grasped a food pellet, brought it into the cage, and consumed it without dropping it. Reaching performance was scored as follows:

$$\frac{Number\;of\;successful\;reaches}{20}\times 100$$

For the foot-fault test, subjects were placed on an elevated ladder and motivated to traverse a 1-m long grid with irregularly spaced openings. The foot-fault score was calculated separately for the contralesional and ipsilesional limbs and performance was assessed daily for three weeks. We observed behaviors for up to 5 minutes in each subject. Two trials were performed three times per week. All behavioral tests were performed under blind conditions.

### MicroPET scan and imaging analysis

We acquired serial micro-PET images in awake animals using FDG-microPET to assess functional change following pyramidotomy. The change in rCGM was evaluated using an ^18^F-FDG microPET/CT scanner (Inveon, Simens Medical Solution, TN, USA) which has a transaxial resolution of 1.4 mm full width at half maximum, with a field of view of 12.7 cm. Each rat in the two groups (sham-operated group, n = 6; pyramidotomy group, n = 6) was scanned three times: the first scan before lesioning (baseline: PL−1), the second scan at post-lesion day (PL) 4, and the third scan at PL7. The rats fasted for 12 hours before scanning and were injected with ^18^F^-^FDG (100mCi/100g) through the tail vein under brief anesthesia with 2% isoflurane. After an uptake period of 30 minutes, animals were anesthetized again by inhalation of isoflurane (2% in 100% oxygen) and placed on the scanning bed with head immobilized. Body temperature was maintained at 36.5 ± 2° C and vital signs including respiration, heart rate, and body temperature were monitored following standard scanning procedure (BioVet, m2m Imaging Corp, and USA). A 25-minute static acquisition with 5 minutes of attenuation correction computed tomography (CT) scan was performed. After scanning, all images were reconstructed using the iterative OSEM3D/MAP algorithm. Imaging analysis was performed the Analysis of Functional NeuroImages (AFNI) package (NIH, Betheda, USA). All acquired images were automatically co-registered to a standard MRI template.[11] Images were normalized to the mean value of whole brain. Finally, spatial smoothing was performed using a 3-D isotropic Gaussian kernel with 1.2 mm full width at half maximum. The 3-D rendering was performed using a MINC tool kit (McConnell Brain Imaging Centre, Montreal Neurological Institute, Montreal).

The region of interest was defined based on the statistical map and several stereotaxic coordinates were chosen to represent the anatomical location of brain activation or deactivation across serial scans.

### Statistical analysis

A group-level linear mixed-effect model was performed with the 3dLME program in AFNI to evaluate the differences between baseline (PL−1), PL4, and PL7 images for the sham-operated and pyramidotomy groups. Statistical maps were corrected and thresholded at the significance level (*p* < 0.001, FDR *q* < 0.05), and then overlaid on the MRI template to show the areas with significant changes in brain activity.

Data analyses were performed with statistical analysis software (OriginPro version 9.1, OriginLab, Northampton, MA, USA). Behavioral data were analyzed with a one-way analysis of variance on the effect of groups and times (*p* < 0.05, Bonferroni corrected for multiple comparisons). All data are represented as the mean ± standard error of the mean (SEM). *p* values < 0.05 were considered significant.

## Results

### Neural tracing of pyramidal fibers

**Fig 1:**
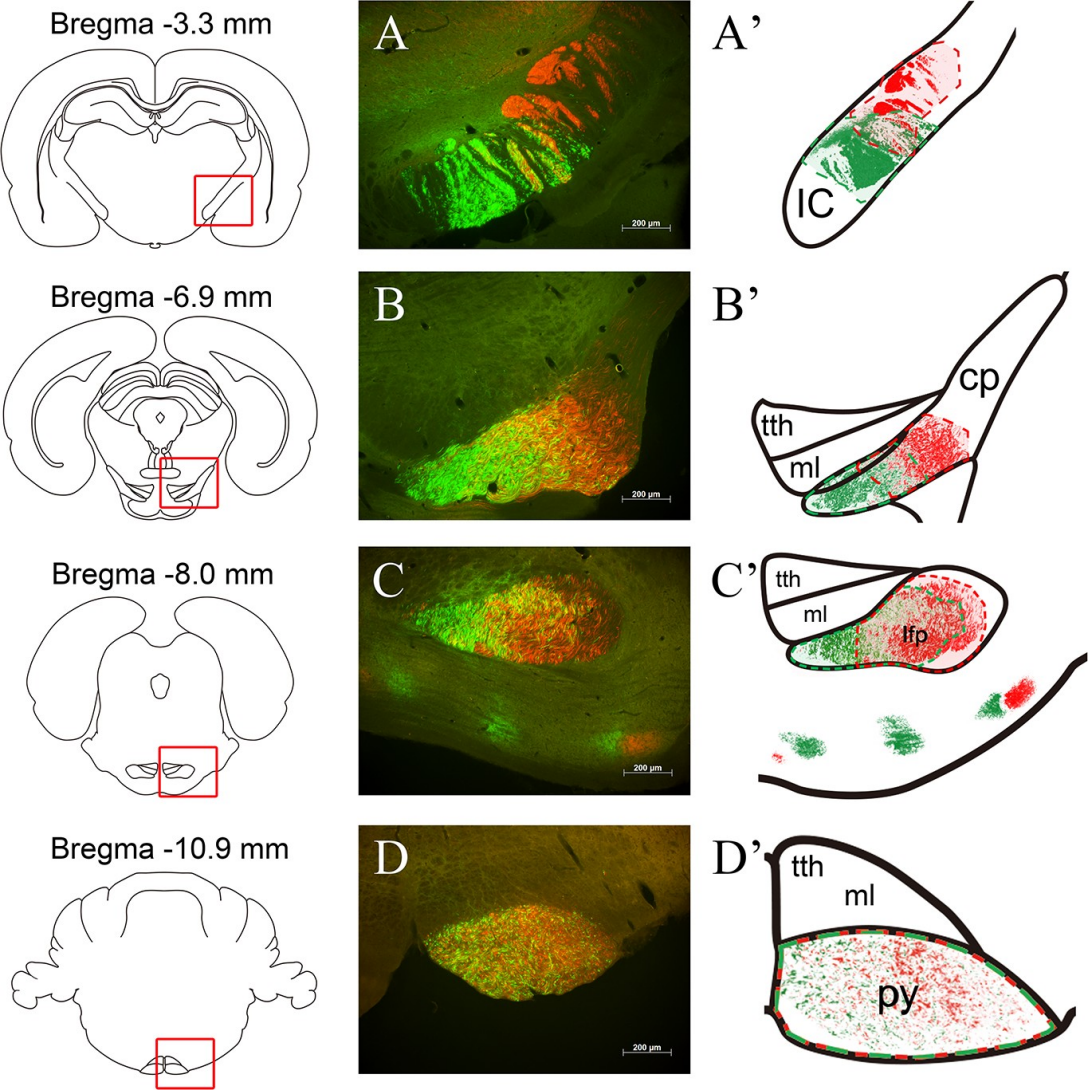
Neural tracing of pyramidal fibers. Serial coronal sections demonstrate the viral expression of forelimb (green) and hindlimb (red) motor fibers at the level of the posterior limb of the internal capsule (A), the cerebral peduncle (B), the longitudinal fasciculus of the pons (C), and the medullary pyramid (D). Right-hand panels are schematic diagrams showing the distribution of forelimb and hindlimb motor fibers mapped onto an anatomical atlas. Numbers indicate the distance from bregma. IC: internal capsule; CP: cerebral peduncle; tth: trigeminothalamic tract; ml: medial lemniscus; lfp: longitudinal fasciculus of the pons; py: pyramid.

We conducted a viral neural tracing study of the course and relative distribution of corticospinal fibers originating from forelimb and hindlimb areas of the motor cortex (n = 9) (Fig 1). At the level of the PLIC, forelimb motor fibers (FMFs) are distributed in the cranio-ventral portion of the PLIC and hindlimb motor fibers (HMFs) are distributed in the caudo-dorsal portion of the PLIC, with a small area of partial overlap. As the pyramidal fibers descend, the degree of overlap between FMFs and HMFs increases. At the level of the cerebral peduncle, FMFs and HMFs maintain their relative ventral and dorsal locations but with greater overlap. At the LFP level, descending fibers from the cerebral peduncle mostly occupy the LFP but some scattered bundles are observed in areas around the LFP. At this level, the degree of mixing between FMFs and HMFs becomes prominent. Finally, after reaching the medullary pyramid, FMFs and HMFs are intermingled and it is difficult to distinguish the relative location of the FMFs and HMFs. However, the density of FMFs (green) is higher than the density of HMFs in the medial portion of the pyramid, indicating that it is essential to destroy the medial portion of the pyramid during pyramidotomy.

### Histological findings after pyramidotomy

**Fig 2:**
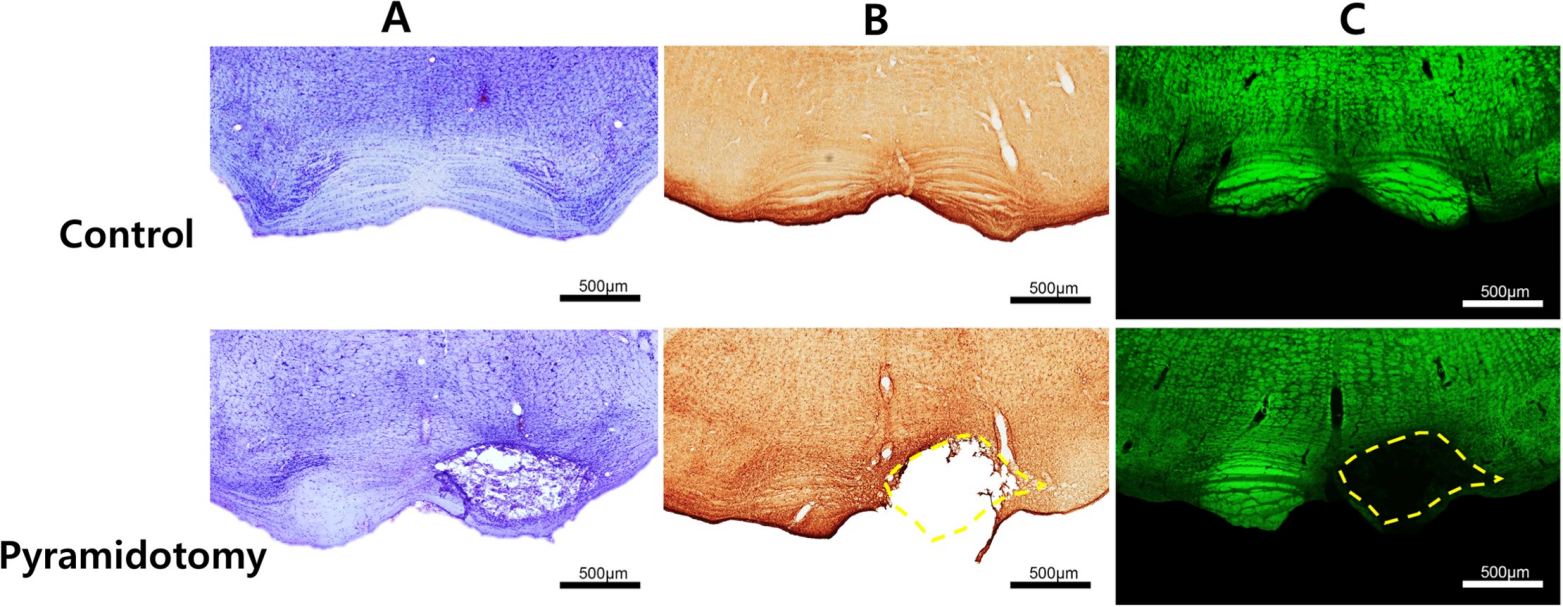
Histological finding of pyramidotomy lesion. Nissel staining (A), and Glial fibrillary acidic protein (B) and myelin (C) immunostaining show the extent of pyramidal infarct lesioning. Upper panel shows the control and lower panel pyramidotomy lesion.

**Fig 3:**
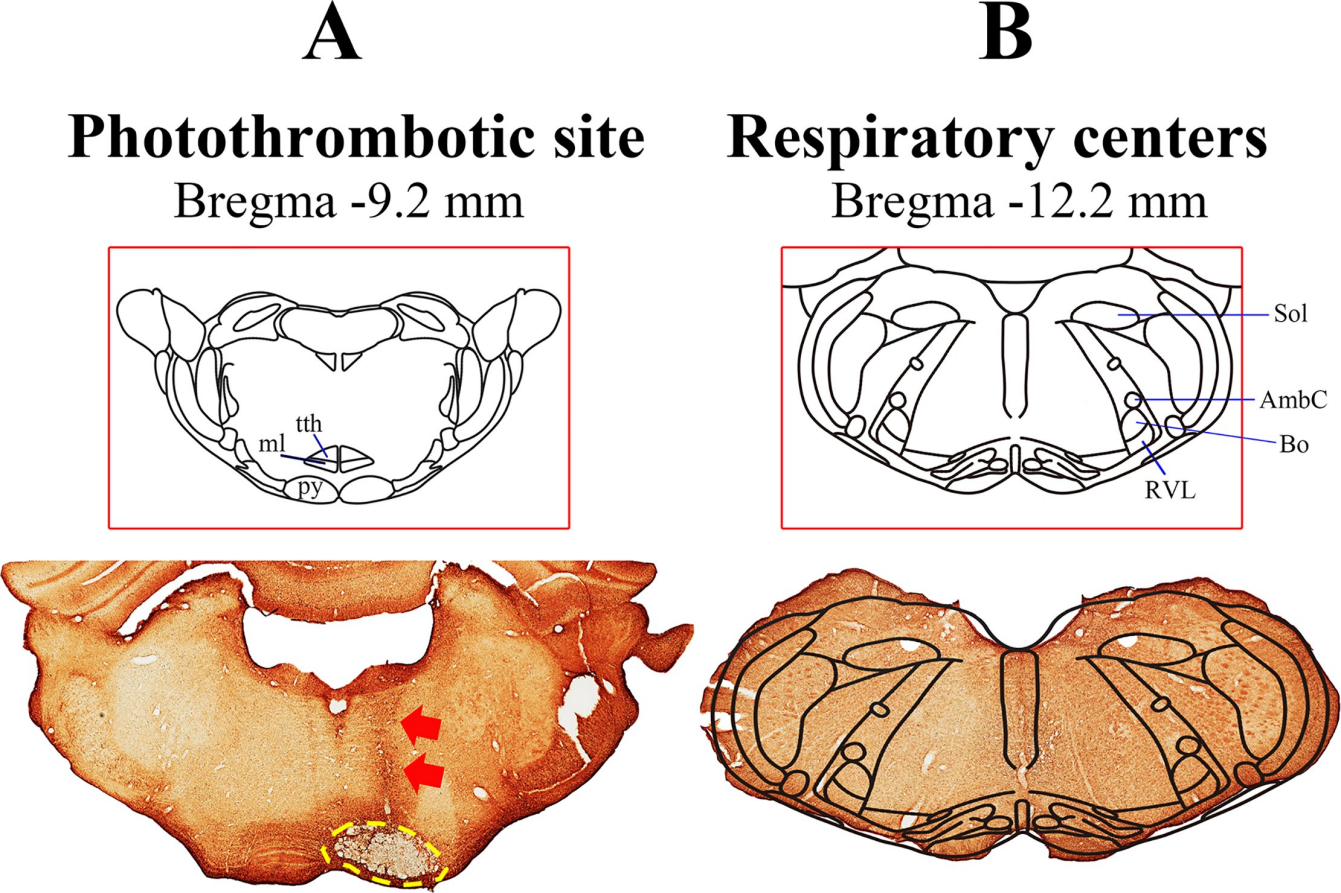
**The location of the photothrombotic lesion (A) relative to the location of respiratory centers in the rat (B).** Red arrows in (A) indicate the track of the optical fiber and the yellow dotted circle indicates the site of the photothrombotic lesion in the medullary pyramid. (B) Respiratory centers located in the lower medulla. tth: trigeminothalamic tract; ml: medial lemniscus; py: pyramid; Sol: nucleus of the solitary tract; AmbC: ambiguus nucleus; Bo: Botzinger complex; RVL: restro ventrolateral reticular nucleus.

Fig 2, S1 Fig and S2 Fig show the pattern of infarct lesions in the unilateral medullary pyramid. At PL1, H&E staining showed that the lesion was well demarcated, with loss of stainability due to intralesional edema and infiltration of macrophages. There was a mild increase in GFAP staining limited to the vicinity of the lesion. NF staining showed a complete loss of axons in the lesion. Iba1 staining showed microglial infiltration along the rim of the lesion. At PL4, the site of the infarct lesion showed a cystic cavity surrounded by reactive gliosis and macrophage infiltration in sections that were stained with H&E, GFAP, and Iba1. Interestingly, at higher magnification we to observed a mild gliotic reaction in the medial portion of the contralateral pyramid (arrow). Perilesional swelling and midline shifts were observed from PL4. The perilesional edema and midline shift persisted at PL7 but GFAP immunoreactivity was slightly decreased at this time point. By PL21, the perilesional edema had subsided and perilesional infiltration by inflammatory cells was also decreased (Fig 2). The infarct volume did not show any statistically significant changes over time. The infarct volume including the area of necrosis and the surrounding demyelination was 2.2 ± 1.2 mm^3^. The infarct lesioning generally spared the neighboring structures. In particular, there was no evidence that respiration-related medullary nuclei (such as the retro-ambiguous and para-ambiguous nuclei, the Botzinger complex, and the nucleus reticularis retroventrolateralis) or cardiac-function-related nuclei (such as the caudoventrolateral reticular nucleus) were influenced by infarct lesioning in our series (Fig 3).

### Behavioral results following pyramidotomy

**Fig 4:**
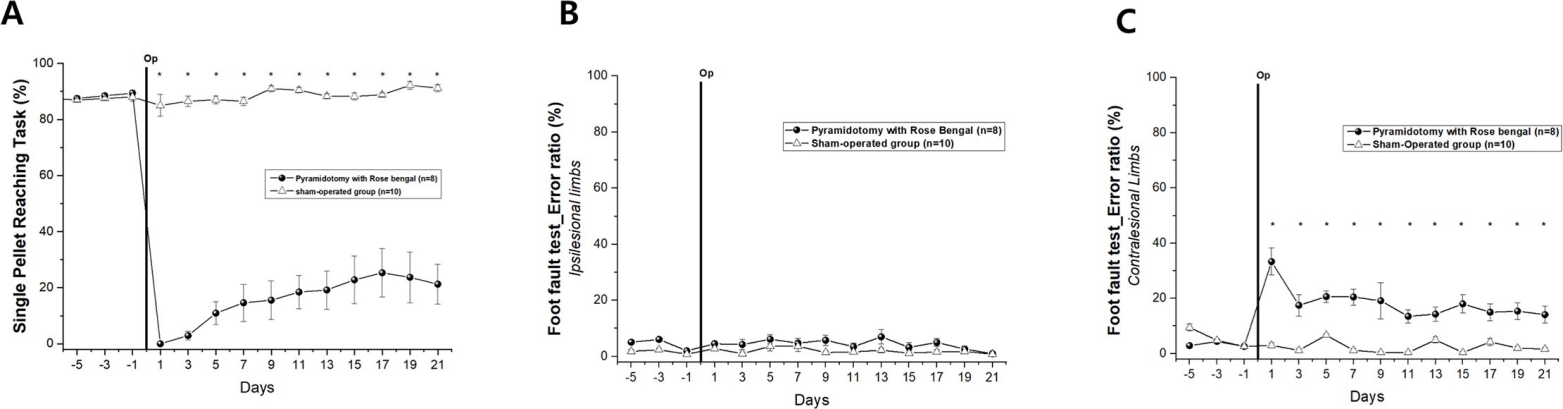
Behavioral evaluation. A. Immediate and persistent decline in performance of the single pellet reaching task following pyramidotomy (*p < 0.005). B & C. Increased error ratio in the foot fault test for the contralesional limb (*p < 0.005) compared with the ipsilesional limb.

Fig 4 shows the behavioral changes before and after infarct lesioning in the medullary pyramid. In the SPRT, the pyramidotomy group showed an immediate deficit in performance in the contralesional forelimb after photothrombotic pyramidotomy compared with the sham-operated group and the motor deficit persisted for the entire observation period (*p* < 0.005) (Fig 4A). The sham-operated group did not show any significant changes from prelesional levels. In the foot-fault test, there was a significant increase in the error ratio for the contralesional limb in the pyramidotomy group compared with the sham-operated group following pyramidotomy (*p* < 0.005), whereas there were no significant differences between pyramidotomy and sham-operated groups for the ipsilesional limb (Fig 4B & 4C).

### MicroPET imaging studies

**Fig 5:**
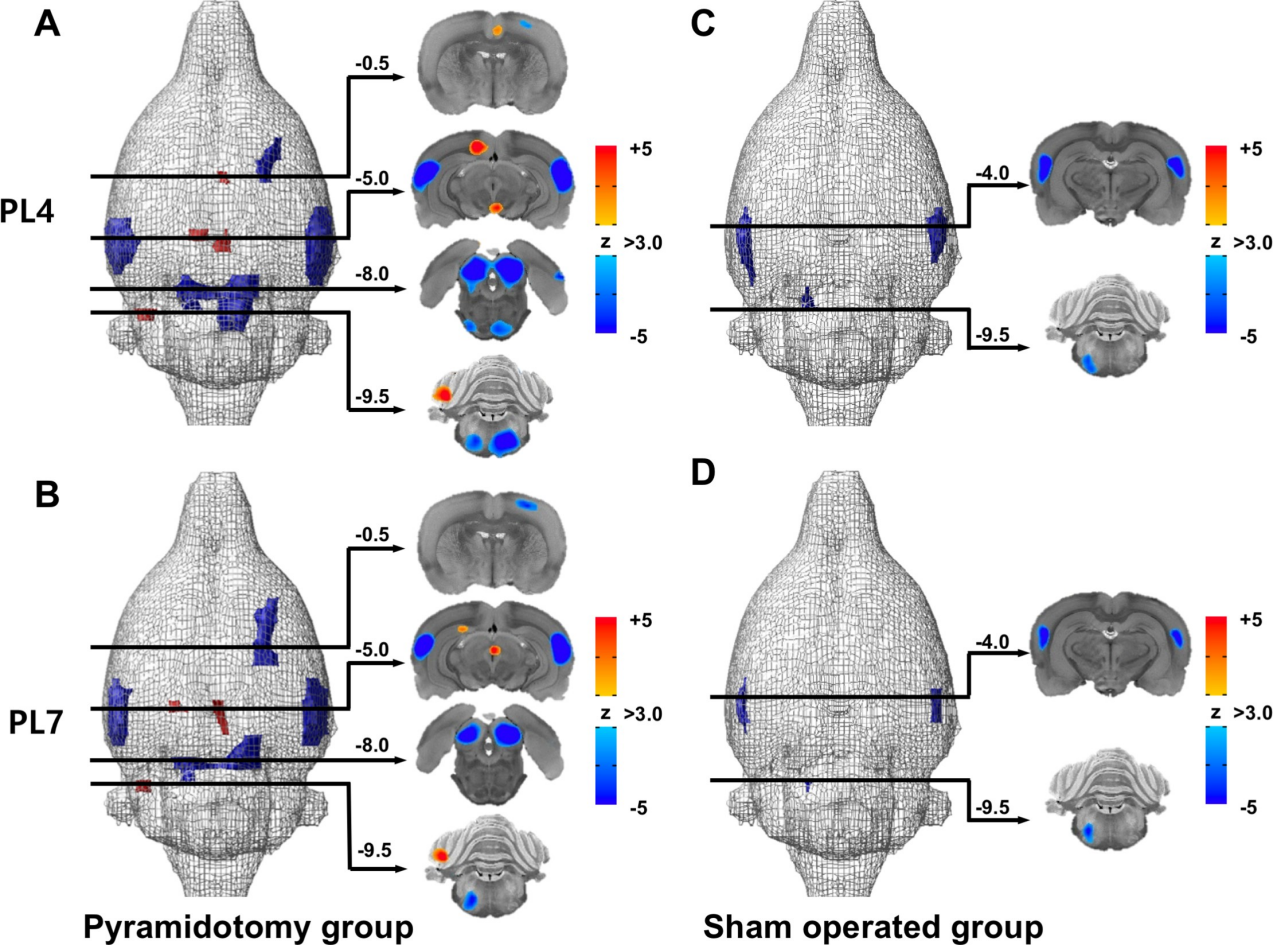
Early longitudinal changes in regional cerebral glucose metabolism after pyramidotomy. The pyramidotomy group (A, B) shows hypometabolism in the contralateral pyramid, the bilateral trapezoid body, inferior colliculi, and auditory cortices, and the ipsilateral sensory cortex at PL4 and PL7 (p < 0.001, false discovery rate q < 0.05). In contrast, hypermetabolism was observed in the contralateral cerebellar hemisphere, the retrosplenial and cingulate cortices, and the ventral tegmentum at PL4, and in the contralateral cerebellar cortex, the periaqueductal gray, and the cingulate motor area at PL7. The sham-operated group (C, D) shows hypometabolism at PL4 and PL7 only in the bilateral trapezoid bodies, inferior colliculi, and auditory cortices.

Fig 5 and S1 Table summarize the early functional effects of pyramidotomy on the entire brain, measured as changes in FDG-PET imaging in pyramidotomy and sham-operated groups at PL4 and PL7. In the pyramidotomy group, a decrease in rCGM was observed at PL4 and PL7 in the contralateral pyramid, in the bilateral trapezoid body, inferior colliculi, and auditory cortices, and in ipsilateral sensory cortex. In contrast, hypermetabolism was observed at PL4 in the contralateral cerebellar hemisphere, the retrosplenial and cingulate cortices, and the ventral tegmentum. At PL7, hypermetabolism was observed in the contralateral cerebellar cortex, the periaqueductal gray, and the cingulate motor area. In the sham-operated group, hypometabolism was observed in the bilateral trapezoid bodies, inferior colliculi, and auditory cortices at both PL4 and PL7. However, other areas did not show hypometabolism or hypermetabolism in comparison with the pyramidotomy group.

### Influence of pyramidotomy on respiration

**Fig 6:**
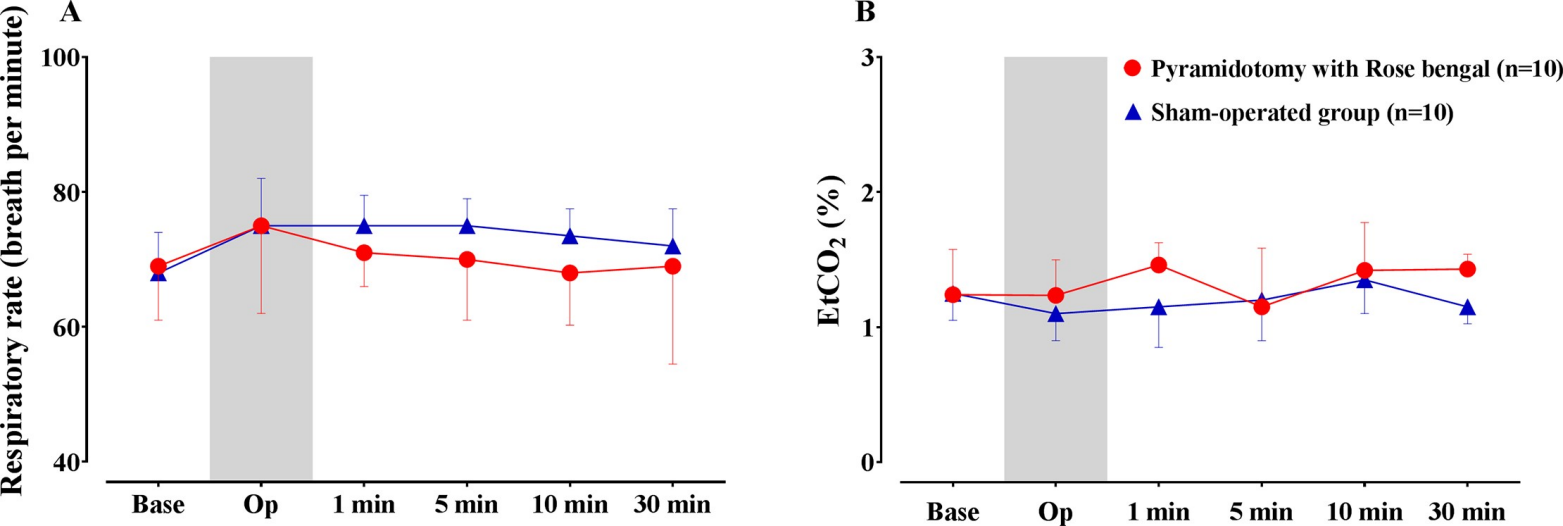
Perioperative monitoring of respiratory rate and end-tidal CO2 concentration (EtCO2). Monitoring of respiratory rate (A) and end-tidal CO_2_ concentration (EtCO_2_) (B) before (base), during (Op), and after photothrombotic lesioning of the medullary pyramid (1, 5, 10, 30 min).

There was no operative mortality in our series. Fig 6 shows the results of physiological monitoring on the respiration during anesthesia: during the prelesioning, lesioning, and postlesioning periods. The respiratory rate was slightly lower in the pyramidotomy group than in the sham-operated group (Fig 6A). However, there was no significant difference between the two groups. Similarly, although the pyramidotomy group had slightly higher EtCO_2_ measurements than the sham-operated group (Fig 6B), the difference was not significant. These results suggest that pyramidotomy does not have a harmful effect on respiratory function.

## Discussion

The present study demonstrates the feasibility of creating a pyramidotomy infarct model by photochemical thrombosis using a dorsally introduced optical fiber. Despite the presence of critical structures in the brain stem through which the optical fiber was introduced, no respiratory disturbances were observed. The viral neural tracing study demonstrated that forelimb and hindlimb motor fibers are distributed evenly in the pyramid, suggesting that complete destruction of the motor fibers is mandatory to achieve a persistent motor deficit. Functional imaging demonstrated the hypometabolism of rCGM in the trapezoid body and the superior olivary nucleus, as well as in related structures, which seems to be induced during the insertion of optical fiber.

The main complication after traditional pyramidotomy with a ventral approach is respiratory difficulty. With a ventral approach, the trachea and cervical muscles covering the clivus must be dissected and retracted; this approach is, therefore, likely to cause tracheal damage and swelling of dissected muscles, often leading to a higher mortality rate in experimental animals. Nonetheless, a posterior transcortical approach has not been attempted because it is believed such an approach would damage the respiratory centers located in the lower medulla[12]. However, in this experiment, we demonstrated that a transcortical approach with a small-diameter optical fiber is safe and effective for generating a circumscribed area of destruction in the medullary pyramid and persistent motor deficits. Models that use a ventral approach tend to show early recovery of motor deficits, but the deficits generated by our model persisted for a relatively long time after pyramidotomy[13–15]. The introduction of the optical fiber left a small track in the parenchyma of the brain; however, this track is quite far from both the dorsal and ventral groups of respiratory centers. Furthermore, respiratory monitoring showed that respiratory rate and EtCO_2_ did not changed during or after photothrombotic pyramidotomy. Consequently, the mortality rate from transcortical pyramidotomy was very low compared with the ventral approach. Therefore, our dorsal transcortical approach to infarct lesioning in the medulla appears to be safer than the traditional ventral approach.

We used the photothrombotic technique because this technique can selectively destroy the CST (white matter)[16]. Previous observations have indicated that photothrombotic infarct lesioning is a good strategy for selectively destroying white-matter tissue without invading the neighboring, critical grey-matter tissue[7, 9]. In a traditional pyramidotomy, a knife is used to remove the medullary pyramid. It is not easy to correctly remove the tissue inside the border of the pyramid even with experienced hands. However, our technique relies on the optical properties of laser light to produce circumscribed destruction within fiber tissues, preserving the neighboring grey matter.

Our neural tracing study revealed that FMFs and HMFs are distributed differently at different anatomical levels. In the internal capsule, FMFs and HMFs are arranged in different locations with only a small amount of overlap between the two fiber bundles. As the FMFs and HMFs descend, the degree of overlapping increases and the two types of fiber are intermixed in the pyramid. However, we observed a higher density of FMFs in the medial side of the pyramid, indicating that the medial portion of the pyramid should be included in the area of infarction to guarantee a persistent motor deficit. Our previous observations indicate that photothrombotic lesioning accurately targeted to the HMF site in the internal capsule can produce permanent motor deficits. Likewise, targeting the medial portion of the medullary pyramid seems to be important to prevent early recovery following pyramidotomy.

This is the first study to determine the functional changes after transcortical medullary pyramidotomy via a posterior approach. We used microPET imaging to assess the influence of pyramidotomy in the whole brain. We identified hypometabolism in the trapezoid body and the superior olivary nucleus, which are located above the medullary pyramid. Given that hypometabolism in these structures occurred in both the pyramidotomy and the sham-operated group, it is plausible that this hypometabolism was caused by damage to these structures during the pyramidotomy procedure. These structures are located above the medullary pyramid and were likely injured when the optical fiber was introduced. In addition, hypometabolism was observed in the bilateral inferior colliculi and auditory cortices in both pyramidotomy and sham-operated groups, suggesting that the injury to the superior olivary nucleus and the trapezoid body result in functional changes in connected structures, such as the inferior colliculi and auditory cortices. We also identified hypometabolism in the contralateral pyramid, which is compatible with the gliotic reaction in the contralateral medullary pyramid that we observed via GFAP staining.

There are several limitations to this model. The dorsal approach to the medullary pyramid has a high probability of injuring the trapezoid body and the superior olivary nucleus because these structures reside near the track of the optical fiber. Injury to these structures may induce auditory problems. Given that medullary pyramidotomy is used to model deficits in motor function, not auditory function, this issue with the dorsal approach may not hamper the usefulness of this pyramidotomy model. In the ventral approach, a knife is used to remove the medullary pyramid. It is not easy to correctly remove the tissue inside the border of the pyramid, even with experienced hands. Considering the paucity of white-matter stroke models, our model may nonetheless be useful for studying the pathogenesis of strokes involving the CST, and mechanisms of recovery. We attempted to destroy the medullary pyramid unilaterally with our technique; however, targeting the medial portion often resulted in the lesion crossing the midline, leading to partial involvement of the contralateral medullary pyramid.

## Supporting information

S1 Fig Pattern of histological changes in unilateral pyramid over the time following pyramidotomy. At PL1, the lesion was well demarcated, with loss of stainability due to intralesional edema and infiltration of macrophages. There was a mild increase in GFAP staining limited to the vicinity of the lesion with complete loss of axons in the lesion (NF) and microglial infiltration along the rim of the lesion (IBa1). At PL4, the site of the infarct lesion showed a cystic cavity surrounded by reactive gliosis and macrophage infiltration in sections that were stained with H&E, GFAP, and Iba1. Interestingly, at higher magnification we to observed a mild gliotic reaction in the medial portion of the contralateral pyramid (arrow). Perilesional swelling and midline shifts were observed from PL4. At PL7, the perilesional edema and midline shift persisted but GFAP immunoreactivity was slightly decreased at this time point.
(TIFF)

S2 Fig Representative histological findings in pyramidotomy group. Photograph shows the infarct lesioning confined to unilateral medullary pyramid without damaging the contralateral pyramid.
(TIFF)

S1 Table Results of significant difference of regional glucose metabolism. The table shows the areas of overlap in peak metabolic changes between baseline scan (PL-1) and post-lesion scans (PL4 and PL7). The representative coordinates indicate distance from the anterior commissure. Results at p <0.001 and false discovery rate (FDR) q <0.05. PL, post-lesion; Cg, cingulate cortex; RSG, retrosplenial granular cortex; D.Hippo, dorsal hippocampus; CBW, cerebellar white matter; VTA, ventral tegmental area; PAG, periaqueductal gray I.Col, inferior colliculus;
(DOCX)
