# Supplementary figures and images for: Transcortical photothrombotic pyramidotomy model with persistent motor deficits
Source: PLoS One. 2018 Dec 31;13(12):e0204842. doi: 10.1371/journal.pone.0204842 (PMC6312246; doi:10.1371/journal.pone.0204842)

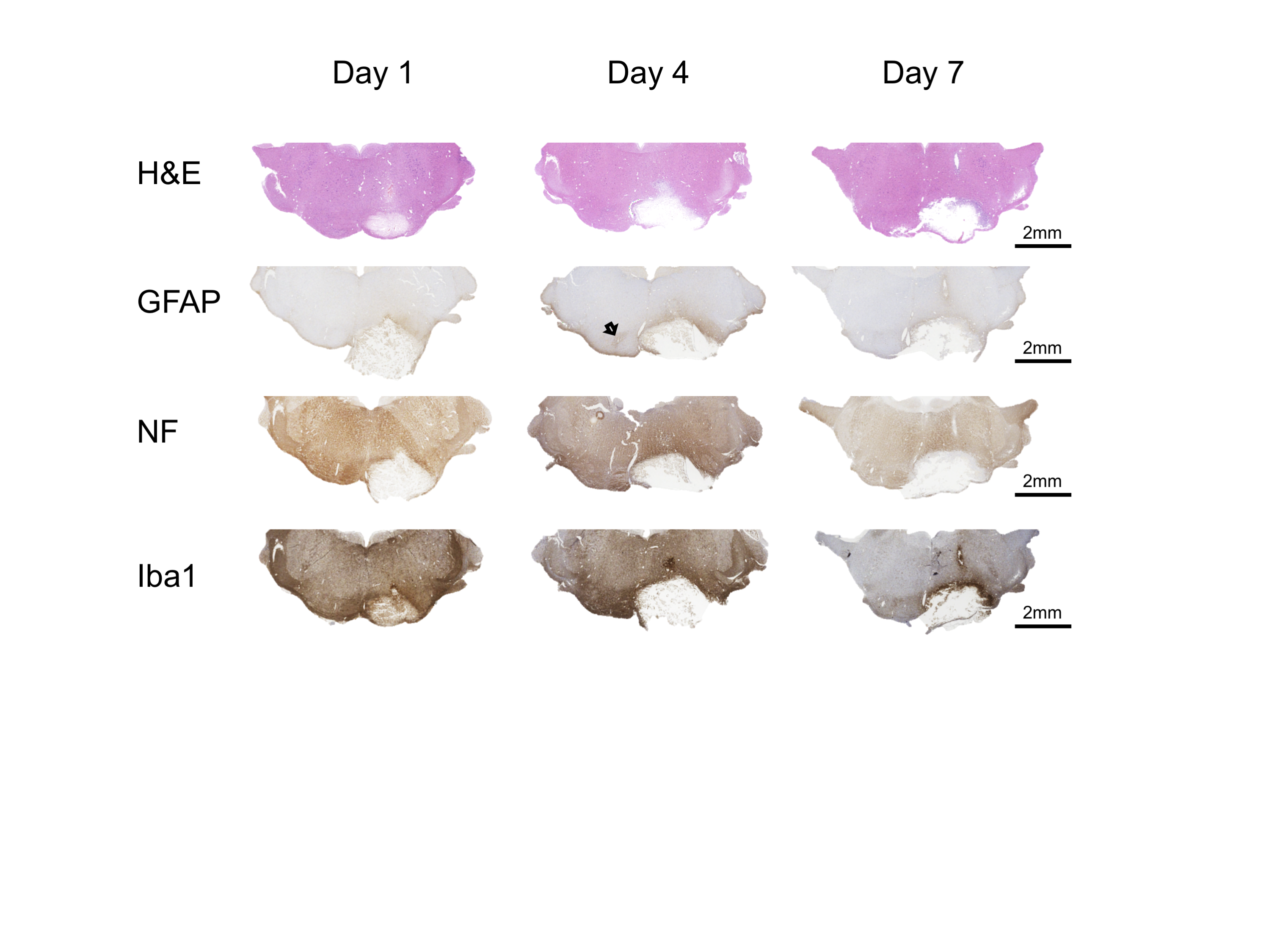

Supplement: S1 Fig — At PL1, the lesion was well demarcated, with loss of stainability due to intralesional edema and infiltration of macrophages. There was a mild increase in GFAP staining limited to the vicinity of the lesion with complete loss of axons in the lesion (NF) and microglial infiltration along the rim of the lesion (IBa1). At PL4, the site of the infarct lesion showed a cystic cavity surrounded by reactive gliosis and macrophage infiltration in sections that were stained with H&E, GFAP, and Iba1. Interestingly, at higher magnification we to observed a mild gliotic reaction in the medial portion of the contralateral pyramid (arrow). Perilesional swelling and midline shifts were observed from PL4. At PL7, the perilesional edema and midline shift persisted but GFAP immunoreactivity was slightly decreased at this time point. (TIFF) [file pone.0204842.s001.tiff]

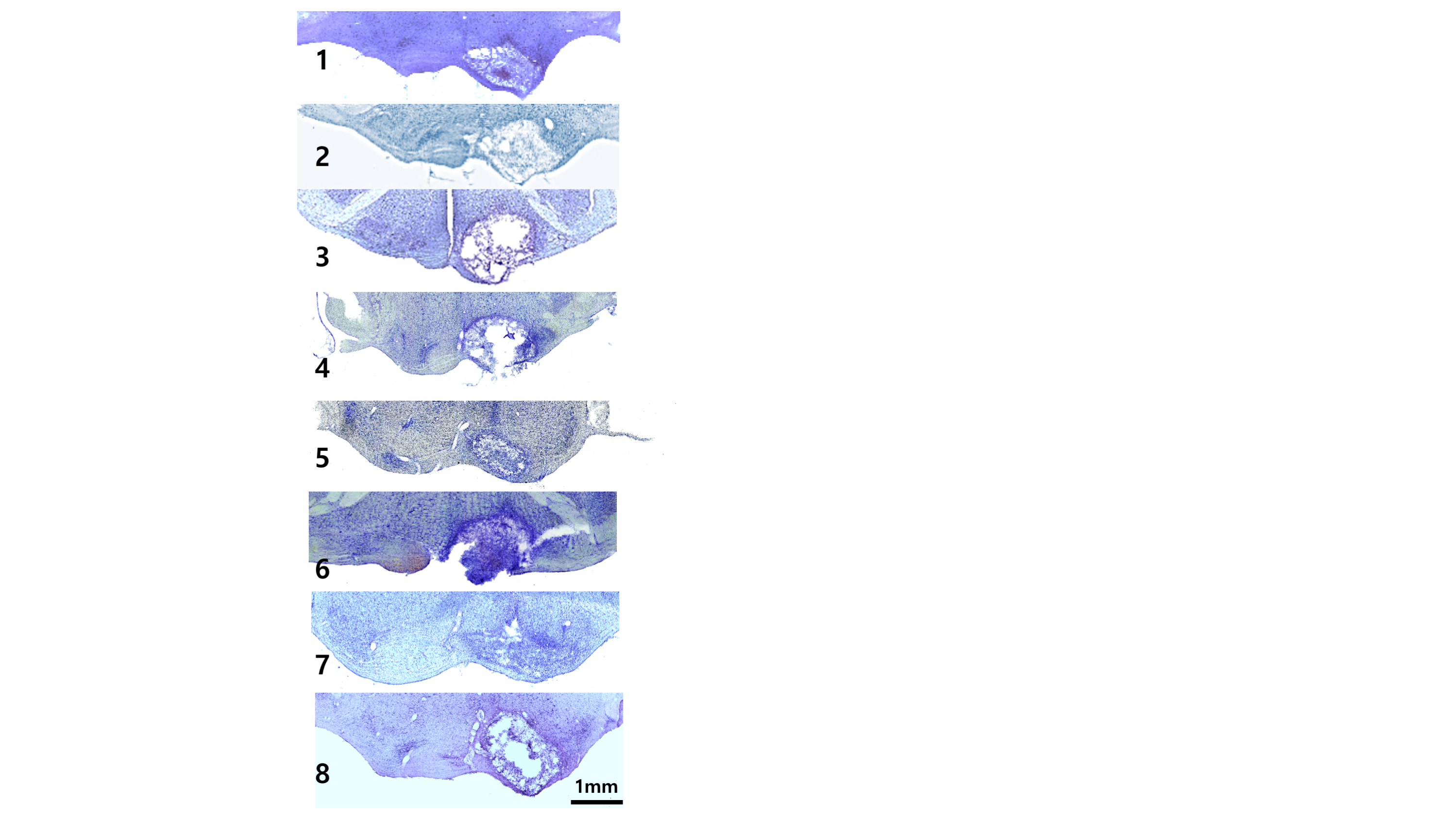

Supplement: S2 Fig — Photograph shows the infarct lesioning confined to unilateral medullary pyramid without damaging the contralateral pyramid. (TIFF) [file pone.0204842.s002.tiff]
