# Supplementary material for: Transcortical photothrombotic pyramidotomy model with persistent motor deficits
Source: PLoS One. 2018 Dec 31;13(12):e0204842. doi: 10.1371/journal.pone.0204842 (PMC6312246; doi:10.1371/journal.pone.0204842)
Supplement: S1 Table — The table shows the areas of overlap in peak metabolic changes between baseline scan (PL-1) and post-lesion scans (PL4 and PL7). The representative coordinates indicate distance from the anterior commissure. Results at p <0.001 and false discovery rate (FDR) q <0.05. PL, post-lesion; Cg, cingulate cortex; RSG, retrosplenial granular cortex; D.Hippo, dorsal hippocampus; CBW, cerebellar white matter; VTA, ventral tegmental area; PAG, periaqueductal gray I.Col, inferior colliculus; (DOCX) [file pone.0204842.s003.docx]

| **Supplementary Table 1. Results of significant difference of regional glucose metabolism** | | | | | | | | | | | | | | | |
| --- | --- | --- | --- | --- | --- | --- | --- | --- | --- | --- | --- | --- | --- | --- | --- |
|  | *Group* |  | *Activation* |  | *Anatomic Area* |  | *Representative Coordinates (mm)* | | |  | *Voxel Size (Z-value)* | | | |  |
|  |  |  |  |  |  |  | X | Y | Z |  | PL4 | PL7 | |  | |
|  | Sham |  | Hypometabolism |  | Pyramid (cont) |  | -2.2 | 9.1 | -2.1 |  | 30(-3.9±0.4) | 14(-4.0±0.6) | |  | |
|  |  |  |  |  | Auditory (ipsi) |  | 6.8 | 4.3 | 2.9 |  | 68(-4.7±1.2) | 37(-4.3±0.7) | |  | |
|  |  |  |  |  | Auditory (cont) |  | -6.6 | 4.8 | 3.0 |  | 108(-5.2±1.8) | 65(-4.6±1.1) | |  | |
|  | Pyramidotomy |  | Hypermetabolism |  | Cg (ipsi) |  | 0.3 | 0.4 | 5.0 |  | 13(3.6±0.2) |  | |  | |
|  |  |  |  |  | RSG (cont) |  | -1.5 | 4.9 | 5.7 |  | 40(4.5±1.2) |  | |  | |
|  |  |  |  |  | D.Hippo (cont) |  | -2.9 | 4.6 | 4.8 |  |  | 13(3.9±0.6) | |  | |
|  |  |  |  |  | VTA |  | 0.4 | 5.0 | -0.2 |  | 17(4.2±0.8) |  | |  | |
|  |  |  |  |  | PAG |  | 0.3 | 5.0 | 2.6 |  |  | 23(4.6±1.1) | |  | |
|  |  |  |  |  | CBW (cont) |  | -5.1 | 9.9 | 3.0 |  | 31(4.4±0.8) | 19(4.2±0.8) | |  | |
|  |  |  | Hypometabolism |  | Motorsensory (ipsi) |  | 3.5 | -0.9 | 5.2 |  | 44(-4.2±0.8) | 73(-4.4±0.8) | |  | |
|  |  |  |  |  | Auditory (ipsi) |  | 7.0 | 5.0 | 3.0 |  | 208(-5.9±1.9) | 176(-5.6±1.9) | |  | |
|  |  |  |  |  | Auditory (cont) |  | -6.9 | 4.9 | 3.0 |  | 150(-6.2±2.9) | 122(-6.1±2.6) | |  | |
|  |  |  |  |  | I. Col (ipsi) |  | 1.7 | 8.0 | 3.9 |  | 115(-6.3±2.4) | 114(-5.5±1.8) | |  | |
|  |  |  |  |  | I. Col (cont) |  | -1.6 | 8.3 | 3.8 |  | 106(-5.4±1.6) | 61(-4.9±1.3) | |  | |
|  |  |  |  |  | Pyramid (ipsi) |  | 1.0 | 9.6 | -2.0 |  | 153(-4.3±0.8) |  | |  | |
|  |  |  |  |  | Pyramid (cont) |  | -2.1 | 8.9 | -1.8 |  | 48(-4.0±0.9) | 32(-4.1±0.6) | |  | |
|  |  | | | | | | | | | | | |  | | |
|  |  |  |  |  |  |  |  |  |  |  |  |  |  | | |
|  |  |  |  |  |  |  |  |  |  |  |  |  |  | | |
